# Perspectives on How Fall Prevention Technologies Can Support Older Adults’ Self-Monitoring Processes

**DOI:** 10.1093/geroni/igab046.478

**Published:** 2021-12-17

**Authors:** Shannon Meija, Sungjae Hong, Aileen Griffin, Tai-Te Su, Jacob Sosnoff

**Affiliations:** 1 University of Illinois, Champaign, Illinois, United States; 2 University of Illinois at Urbana-Champaign, Aurora, Illinois, United States; 3 University of Illinois at Urbana-Champaign, Champaign, Illinois, United States; 4 School of Health Professions, University of Kansas Medical Center, Kansas City, Kansas, United States

## Abstract

Fall risk increases as older adults lose the functional resources necessary to maintain balance while completing everyday activities. As functional resources often decline gradually with age, momentary deficits may not be apparent until after a fall occurs. Mobile fall prevention technologies could support older adults in self-monitoring their ability to safely navigate their environments. In this paper we present perspectives on self-monitoring and feedback in a sample of older adults (n = 20, 50% female, age 65+) who had self-assessed their balance via a smartphone for 30 consecutive days. Thematic analysis of semi-structured interviews showed that fall history differentiated a) participants’ awareness of day-to-day variation in functional ability; b) trust in the accuracy of self-monitoring; and c) imaginations of what types of feedback a mobile fall prevention technology should provide. Insight on older adults’ internal self-monitoring processes and guidelines for feedback design are discussed.

